# Giant peritoneal loose body and its protein composition: a case report

**DOI:** 10.1186/s12894-024-01425-8

**Published:** 2024-02-17

**Authors:** Weicong Sang, Yang Li, Xiaoping Hong, Haihong Qu, Rujian Zhu, Qingtong Yi

**Affiliations:** 1 Shanghai Pudong Hospital, Fudan University Pudong Medical Center, Shanghai, China; 2 Nursing Department of Shanghai Pudong Hospital, Fudan University Pudong Medical Center, Shanghai, China

**Keywords:** Peritoneal loose body (PLB), Protein components, Collagens, Histochemical stain

## Abstract

Peritoneal loose body (PLB) is a kind of lesions located in the abdominal cavity or pelvic cavity, which is rare and difficult to diagnose. The diameter of PLB is mostly 0.5–2.5 cm. Most PLBS are asymptomatic. Here we reported a case of giant PLB in the pelvis and analyzed its structure and protein composition. Surgical exploration revealed a white oval mass (4.5*4*3 cm) in the pelvic cavity. After the mass was removed, the symptoms of hematuria disappeared and the patient was discharged on the second postoperative day. Histochemical staining showed that PLB was mainly composed of collagen and scattered calcification. The protein components of PLB were detected by proteome analysis, and a variety of proteins related to collagen deposition and calcification were identified in PLB.

## Introduction

Peritoneal loose bodies (PLB), also referred as “peritoneal mice”, are lesions of various sizes occurring in the abdominal cavity, which are rare in clinical practice but sometimes observed during exploration laparotomy [1, 2]. The most prominent feature of PLB is its smooth surface, no adhesion to surrounding organs, and the position of the mass in the abdominal cavity can alter with the change of body position [3]. At present, the pathogenesis of PLB is not clear. At present, a more reliable theory of the cause of PLB is that the necrosis and exfoliation of fat tissue such as intestinal lipoma and omentum majus, which is accompanied by the deposition of serum protein in the abdominal cavity for a long time, and the central part gradually becomes saponified and calcified, and finally forms a smooth egg-like mass with the surface [4–6]. It has been reported that PLB are more common in males, and common PLBS are mostly between 5 and 20 mm in diameter, and PLB larger than 50 mm are very rare [7]. The symptoms caused by PLB are diverse, such as abdominal pain, urgency of urination, intestinal obstruction, etc. [8–10]. It is easy to be misdiagnosed as benign or malignant abdominal tumors, tuberculous granuloma, etc. [11]. We report a case of a 67-year-old male patient who was found to have a peritoneal loose body by laparoscopy, measuring approximately 4.5*4*3 cm.

## Individual patient data

**Fig. 1:**
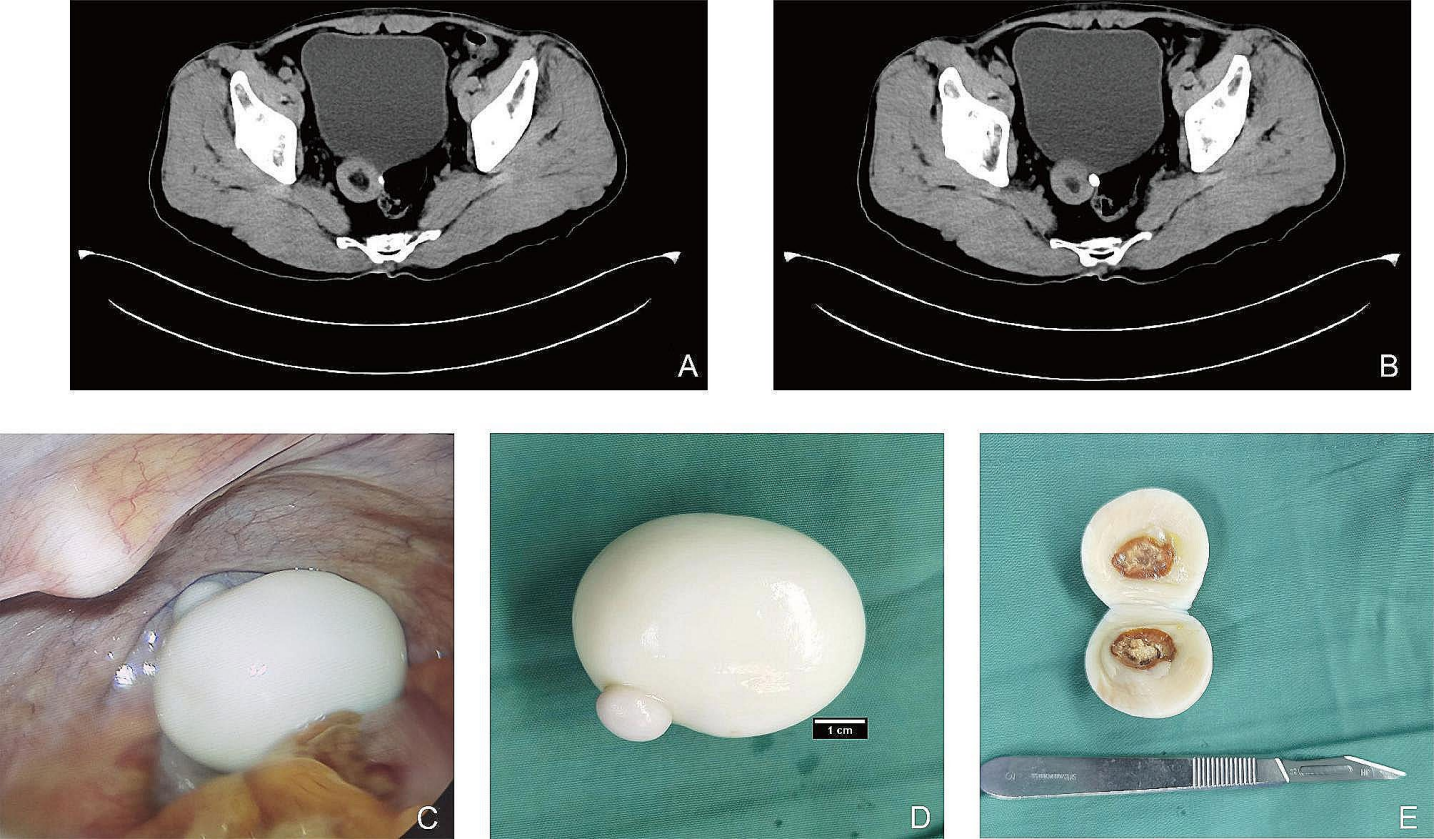
**A**&**B**: Abdominal CT showed a 4*3.8 cm mass in the right space between bladder and rectum; **C**: Morphology of the lesion under laparoscopy. **D**: The lesion is a white oval mass with a size of about 4.5*4*3 cm, which is slippery, tough, no adhesion with the surrounding tissue. **E**: The lesion had a calcified center

A 67-year-old male patient was admitted to the hospital due to recurrent urethral bleeding for 4 days. He had no urinary frequency, urgency or pain, abdominal pain or diarrhea, nausea and vomiting, fever, fatigue or other discomfort. Abdominal CT showed a 4*3.8 cm mass in the right space between bladder and rectum (Fig. 1A&B). The patient’s gross hematuria could not identify the cause, and CT showed a pelvic mass, which was highly likely to be a pelvic tumor. Therefore, we decided to perform laparoscopic pelvic lesion resection and cystoscopy. During the operation, an egg-like mass was found at the lowest position of the abdominal cavity (Fig. 1C), which was a white oval mass with a size of 4.5*4*3 cm. The surface was smooth and the texture was tough, and there was no adhesion with the surrounding tissue. A bean-sized protuberant was seen on the surface of the mass, round and hard in texture (Fig. 1D). The mass, about 1 cm in diameter, showed a calcified center (Fig. 1E). Histologically, it was composed of collagens and calcified tissues without normal cellular components. Cystoscopy revealed no neoplasms or stones in the bladder. The bilateral ureteral orifice was clearly visible, and the color of urine was clear. The hematuria disappeared after the mass was removed, and the patient was discharged on the second day after the operation. The patient’s cystoscopy was normal, and there was no frequent urination, urgency and pain, no abdominal adhesions in the abdominal cavity. Therefore, we speculated that the patient’s hematuria may be caused by peritoneal loose body.

## Histochemical stain

**Fig. 2:**
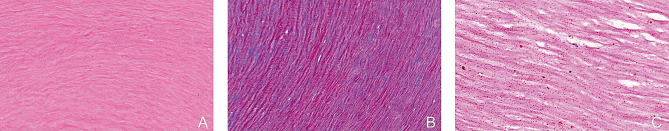
**A**: HE staining showed that the lesion had no obvious cellular structure; **B**: Masson staining showed that the lesion was composed of neatly arranged collagens; **C**: Von Kossa staining showed that scattered calcifications in the lesion

 Histologically, HE staining, Masson staining and Von Kossa staining were performed. HE staining showed that the lesions were fibrous tissue without cellular structure and arranged neatly.(Fig. 2A) The blue part of Masson staining suggests that the lesion tissue contains abundant and neatly arranged collagen fibers, and the red part may be keratin.(Fig. 2B) Von Kossa staining suggested that the lesion contained scattered calcification. (Fig. 2C)

## Proteome analysis

Liquid chromatography-mass spectrometry was used to analyze the protein composition in the lesions, and a total of 492 proteins were identified, of which 42 proteins were related to collagen fibers and adhesion. (Table 1) Among them, Asporin (ASPN) can bind with collagens and calcium and may induce the calcification of collagen [12].

**Table 1:** Mass spectrometric analysis found proteins related to collagens and adhesion

Entry	Gene names	Entry	Gene names	Entry	Gene names
D9ZGF2	COL6A3	P02452	COL1A1	P22352	GPX3 GPXP
A0A384MDM4	Epididymis secretory sperm binding protein	P02671	FGA	P02461	COL3A1
A0A384P5H7	COL6A1 hCG_401279	V9HWF6	HEL-S-153w	P05997	COL5A2
A0A384MDP3	Epididymis secretory sperm binding protein	Q96IY4	CPB2	A0A024R972	Laminin, gamma 1 (Formerly LAMB2), isoform CRA_a
A0A0S2Z4Q2	TGFBI	A0A024RAB6	HSPG2 hCG_1981506	A0A172Q3A0	Fibroblast activation protein
D6RGG3	COL12A1	P08697	SERPINF2 AAP PLI	A0A4D5RAH1	Annexin
Q6FH10	DCN DKFZp686J19238 hCG_24110	P19652	ORM2 AGP2	A0A182DWH7	Selenoprotein P
D9ZGG2	VTN	Q59EE6	Latent transforming growth factor beta binding protein 2 variant	Q5U0B9	Stem cell growth factor; lymphocyte secreted C-type lectin
P01023	A2M CPAMD5 FWP007	P05546	SERPIND1 HCF2	Q9BUM6	COL6A2 protein (Collagen, type VI, alpha 2)
P00747	PLG	B7ZAJ4	LOX	A0A024QZ34	Microfibrillar-associated protein 4, isoform CRA_a
A0A140VJJ6	Testicular tissue protein Li 70	Q6P528	ASPN	Q5M8T4	Connective tissue growth factor
P02452	COL1A1	M1LAK4	OLFML3 hCG_38642	Q9NR99	Matrix-remodeling-associated protein 5
A0A024R944	SERPINC1 hCG_23693	A0A024R971	FMOD hCG_24329	H0YMD0	Annexin
A0A384MDU2	COL1A2 hCG_1686428	A0A024R6R4	MMP2 hCG_24148	G3XAI2	Laminin subunit beta 1

## Discussion

Peritoneal loose bodies, also known as peritoneal mice, is a very rare abdominal lesion, which is often seen in abdominal surgery or autopsy [13, 14]. Zhang et al. studied 22 peritoneal loose bodies and found that this lesion was more common in men aged 50 to 70 years, with a male to female incidence ratio of 18:4 [6]. As for the source of PLB, it is currently speculated that the most likely cause is chronic saponification and calcification of tissues such as intestinal lipoma and greater omentum [15]. One possible process of PLB formation is that a portion of the omentum becomes twisted and the blood supply is cut off, followed by saponification and calcification of the adipose tissue with continuous recruitment of collagens and proteins. Finally, this part of necrotic omentum tissue is shed and PLB is formed. In addition to a larger calcification center, another smaller calcification center was seen on the surface of the PLB in this case. Based on the theory of PLB formation, we believe that this smaller calcification center has the potential to form another PLB at the original site.

The symptoms caused by Peritoneal loose body are various, and most of them are related to the location and movement in the abdominal cavity. The main symptom of the patient in our report was hematuria, which has not been reported in the literature before. Combined with the CT findings of this patient and the location of the PLB in the abdominal cavity, we considered that hematuria might be caused by PLB compression of the bladder or ureterovesical entrance. After surgery, the patient’s hematuria resolved.

We confirmed by histochemical staining that PLB was mainly composed of collagen and protein, lacking normal cellular architecture. The protein composition of PLB was examined by proteomic analysis. In the protein composition of PLB, we found a large number of proteins related to collagens formation, up to 42 proteins. Among them, Asporin (ASPN) can promote collagen binding to proteins and may induce collagen calcification [12]. ASPN is highly expressed in the omentum, and given the important role of the omentum in PLB formation, we suggest that ASPN plays a crucial role in PLB formation. Laminin plays an important role in the formation of collagen network [16]. Connective tissue growth factor (CTGF) can induce collagen deposition and promote collagen formation [17]. Various collagen isoforms, such as COL5A2, COL1A1, COL12A1, COL6A3 and COL3A1, play important roles in collagen deposition and collagen network formation [18–20]. Several different collagen-related proteins are involved in the formation of PLB. In addition, Testicular tissue protein Li 70, epididymo-associated protein and testicular tissue sperm-binding protein were also found in this PLB. We speculate that it may be related to the formation of PLB in the pelvic cavity in this patient. We suggest that the protein composition of PLB may vary depending on the site of formation in the human body.

Our article reported the diagnosis and treatment of a case of giant peritoneal loose body and discussed the causes and symptoms of peritoneal loose body. We first reported the possibility of hematuria caused by giant PLB in the pelvis. The CT findings of PLB are often misdiagnosed as tumors. CT examination showed that the lesions were round or oval, with clear and smooth boundary and calcification in the middle, and enhanced CT showed no blood supply. More importantly, the PLB may shift greatly with the change of body position. Therefore, when an unexplained mass in the abdominal or pelvic cavity is found in CT examination, the patient can change the position for many times. When the mass moves with the change of position, the possibility of PLB should be considered. At present, the exact diagnosis of PLB still needs to be completed by abdominal exploration. Laparoscopic exploration is still recommended. Laparoscopic exploration can not only reduce the pain caused by surgery, but also shorten the length of hospital stay.

Giant peritoneal loose body is a very rare disease and is often found incidentally during abdominal exploration or autopsy. The diagnosis of PLB should be considered when the abdominal mass of unknown origin changes with the change of body position by CT examination.

## Data Availability

Our data was fully available for scientific research.

## Data availability

The data and materials in this research are available for scientific research.

## References

[1] Matsubara K (2017). Laparoscopic extraction of a giant peritoneal loose body: case report and review of literature. Int J Surg Case Rep.

[2] Wen Y (2021). Peritoneal loose body presenting as a hepatic mass: a case report and review of the literature. Open Med (Wars).

[3] Allam T (2013). Peritoneal mouse as detected on (18)F-FDG PET-CT. Front Oncol.

[4] Huang Q (2017). Two giant peritoneal loose bodies were simultaneously found in one patient: a case report and review of the literature. Int J Surg Case Rep.

[5] Ghosh P (2006). Peritoneal mice implicated in intestinal obstruction: report of a case and review of the literature. J Clin Gastroenterol.

[6] Zhang H (2015). Giant peritoneal loose body in the pelvic cavity confirmed by laparoscopic exploration: a case report and review of the literature. World J Surg Oncol.

[7] Lee KH, Song MJ, Park EK (2017). Giant Peritoneal Loose body formation due to Adnexal Torsion. J Minim Invasive Gynecol.

[8] Obaid M, Gehani S (2018). Deciding to remove or leave a peritoneal Loose body: a Case Report and Review of Literature. Am J Case Rep.

[9] Shepherd JA (1951). Peritoneal loose body causing acute retention of urine. Br J Surg.

[10] Bhandarwar AH (1996). Acute retention of urine due to a loose peritoneal body. Br J Urol.

[11] Elsner A (2016). Symptomatic giant peritoneal loose body in the pelvic cavity: a case report. Int J Surg Case Rep.

[12] Yang W (2022). Comprehensive bioinformatics analysis of susceptibility genes for developmental dysplasia of the hip. Intractable Rare Dis Res.

[13] Teklewold B (2019). A giant egg-like symptomatic Loose body in the peritoneal cavity: a Case Report. Ethiop J Health Sci.

[14] Ariaya A, Ahmed M, Mindaye ET (2021). Incidental peritoneal loose body in a polytrauma patient: the unnoticed scenario: a case report. Int J Surg Case Rep.

[15] Kavanagh DO et al. *Laparoscopic retrieval of a peritoneal mouse* Case Rep Med, 2010. 2010. 10.1155/2010/624825 PMC2946616 20885943

[16] Imai M (2015). Three-dimensional morphogenesis of MDCK cells induced by cellular contractile forces on a viscous substrate. Sci Rep.

[17] Atazadegan MA et al. *The effects of Medicinal plants and Bioactive Natural compounds on Homocysteine*. Molecules, 2021. 26(11). 10.3390/molecules26113081 PMC8196702 34064073

[18] Chen G (2020). Foxf2 and Smad6 co-regulation of collagen 5A2 transcription is involved in the pathogenesis of intrauterine adhesion. J Cell Mol Med.

[19] Soták M et al. *Healthy subcutaneous and omental adipose tissue is Associated with High expression of Extracellular Matrix Components*. Int J Mol Sci, 2022. 23(1). 10.3390/ijms23010520 PMC8745535 35008946

[20] Liu X (2015). Muscle Transcriptional Profile based on muscle Fiber, mitochondrial respiratory activity, and metabolic enzymes. Int J Biol Sci.

